# Animal Models for Anorexia Nervosa—A Systematic Review

**DOI:** 10.3389/fnhum.2020.596381

**Published:** 2021-01-20

**Authors:** Sophie Scharner, Andreas Stengel

**Affiliations:** ^1^Department for Psychosomatic Medicine, Charité Center for Internal Medicine and Dermatology, Berlin Institute of Health, Charité-Universitätsmedizin Berlin, Humboldt-Universität zu Berlin, Berlin, Germany; ^2^Department of Psychosomatic Medicine and Psychotherapy, University Hospital Tübingen, Tübingen, Germany

**Keywords:** activity, brain-gut axis, food restriction, psychosomatic, reward, stress

## Abstract

Anorexia nervosa is an eating disorder characterized by intense fear of gaining weight and a distorted body image which usually leads to low caloric intake and hyperactivity. The underlying mechanism and pathogenesis of anorexia nervosa is still poorly understood. In order to learn more about the underlying pathophysiology of anorexia nervosa and to find further possible treatment options, several animal models mimicking anorexia nervosa have been developed. The aim of this review is to systematically search different databases and provide an overview of existing animal models and to discuss the current knowledge gained from animal models of anorexia nervosa. For the systematic data search, the Pubmed—Medline database, Embase database, and Web of Science database were searched. After removal of duplicates and the systematic process of selection, 108 original research papers were included in this systematic review. One hundred and six studies were performed with rodents and 2 on monkeys. Eighteen different animal models for anorexia nervosa were used in these studies. Parameters assessed in many studies were body weight, food intake, physical activity, cessation of the estrous cycle in female animals, behavioral changes, metabolic and hormonal alterations. The most commonly used animal model (75 of the studies) is the activity-based anorexia model in which typically young rodents are exposed to time-reduced access to food (a certain number of hours a day) with unrestricted access to a running wheel. Of the genetic animal models, one that is of particular interest is the *anx/anx* mice model. Animal models have so far contributed many findings to the understanding of mechanisms of hunger and satiety, physical activity and cognition in an underweight state and other mechanisms relevant for anorexia nervosa in humans.

## Introduction

Anorexia nervosa is an eating disorder characterized by an intense fear of gaining weight and a distorted body image which usually leads to low caloric intake and hyperactivity (American-Psychiatric-Association, 2013). Women and girls are nine-times more often affected than men or boys (Nagl et al., 2016). Frequent comorbidities are depression, obsessive-compulsive disorder and suicidality (Treasure et al., 2015). Somatic sequelae are various: electrolyte abnormalities, osteoporosis, cardiac abnormalities, or brain atrophy, to name just a few (Ghadirian et al., 1993). These comorbidities and somatic complications are one of the reasons why anorexia nervosa is the psychiatric disorder with the highest mortality rate (Arcelus et al., 2011). The underlying mechanism and pathogenesis of anorexia nervosa is still poorly understood. The treatment options are still quite limited to mainly nutritional support and psychotherapy, and treatment success is hampered by high relapse rates (Zipfel et al., 2015).

Scientists often try to develop animal models of a disease to understand basic neurobiological processes that are either conserved across species or if not—at least provide conceptual insight on the subject. In order to learn more about the underlying pathophysiology of the eating disorder anorexia nervosa and to find further possible treatment options, several animal models mimicking anorexia nervosa have been employed (Mequinion et al., 2015a). Some of these animal models have been developed and some have been discovered out of coincidence. The aim of this review is to systematically search different databases and provide an overview of existing animal models and to compare them with each other. The purpose is also to discuss advantages and disadvantages to identify which might be the most suited model. We will also highlight the current knowledge gained from the different animal models concerning anorexia nervosa. Lastly, we also discuss gaps in knowledge to highlight where more research is necessary.

## Methods

**Figure 1:**
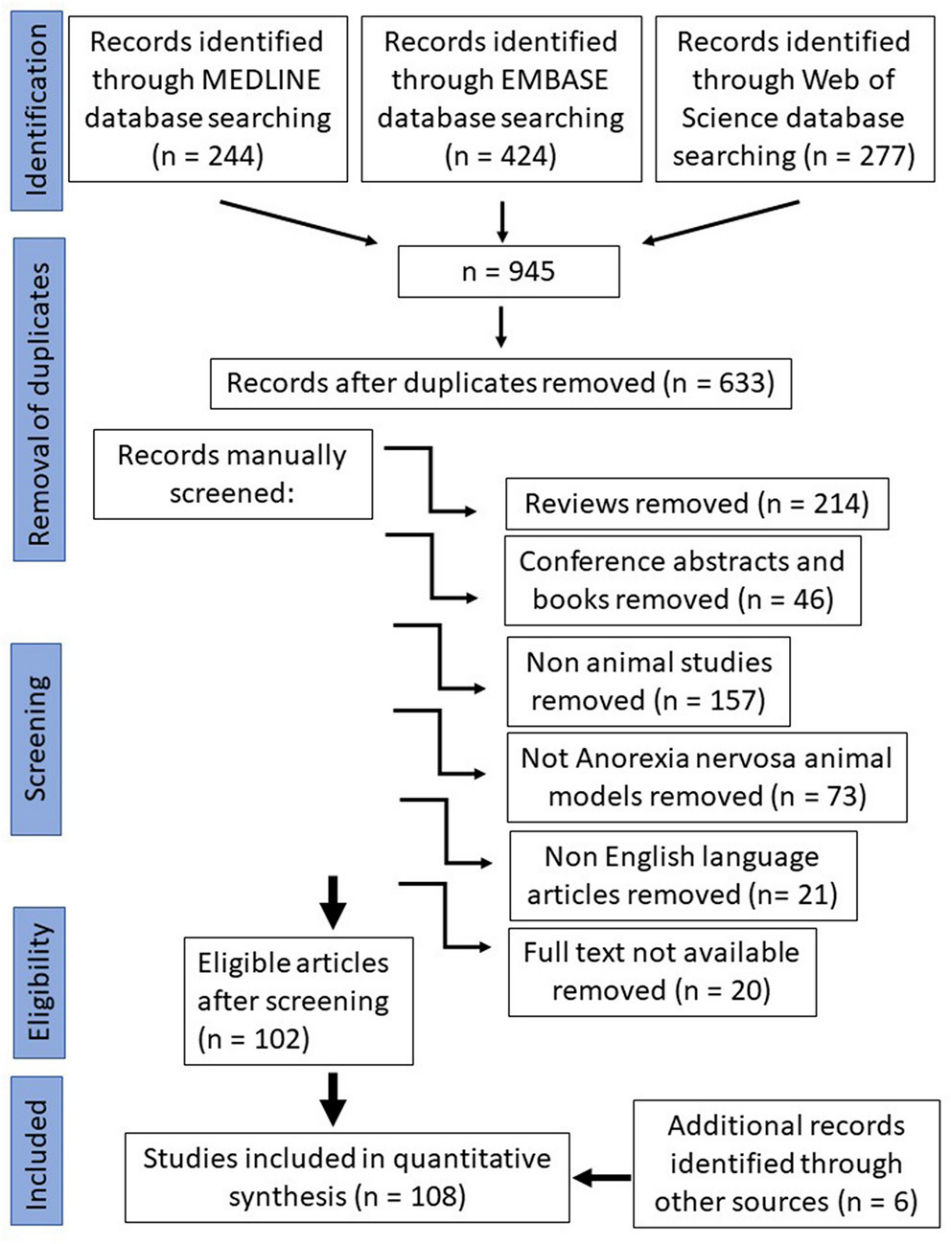
Prisma flow chart.

For the systematic data search, the three commonly used scientific databases, Pubmed—Medline database, Embase database and Web of Science database, were searched using the following search terms: “Anorexia nervosa” and “Animal model”. The search was performed on April 2nd, 2020. The search provided 945 results. Afterwards, duplicates were removed which were around a third (312). Selection criteria applied were original publications (reviews *n* = 214, conference abstracts and book chapters *n* = 46 were removed), animal studies (human studies *n* = 157 were removed), animal studies of anorexia nervosa (animal studies of other diseases than anorexia nervosa *n* = 73 were removed), full text availability (20 papers were removed) and English language (different language than English papers were removed *n* = 21). During the manual screening all publications were selected which study animal models that mimic the eating disorder anorexia nervosa. After selection, 108 publications were included in this systematic review (Figure 1).

## Results

The initial search of the three scientific databases that we chose gave 945 results. About a third of the papers were duplicates (312 papers), which shows that these three chosen databases do have some overlap in results. The 633 records that were left after removal of duplicates were manually screened for eligibility according to our criteria (Figure 1). The resulting 108 studies that we included in our quantitative synthesis were then analyzed for species, animal model protocol and important findings (Table 1).

**Table 1:** Overview of the 108 selected studies in alphabetical order.

**References**	**Year**	**Species**	**Model**	**Strain**	***N***	**Methods: Anorexia protocol**	**Topic and important findings**
Achamrah et al. (2016b)	2016	Mice	ABA	C57Bl/6	72 m	Progressive limited food access from 6 h/day (day 6) to 3 h/day (day 9), wheel 24 h	Access to running wheel during refeeding from ABA in mice improves body composition, intestinal hyperpermeability and behavior
Achamrah et al. (2016a)	2017	Mice	ABA	C57Bl/6	24 m 16 f	Progressive limited food access from 6 h/day (day 6) to 3 h/day (day 9), wheel 24 h	Sex differences in response to ABA: male mice more susceptible and higher mortality rate
Adams et al. (2009)	2009	Rats	Activity	SD	40 m	Rats have running wheel access and on third day get injection, voluntary food reduction	Drug treatment: Chlorpromazine prevents running induced feeding suppression
Altemus et al. (1996)	1996	Rats	ABA	SD	66 f	1.5 h food, 22.5 h wheel	Drug treatment: Fluoxetine prevented ABA, imipramine no effect; PCPA aggravated ABA
Aoki et al. (2012)	2012	Rats	ABA		24 f	1 h food, 24 h wheel	Brain changes in adolescent ABA: more GABA receptors in hippocampus
Aoki et al. (2014)	2014	Rats	ABA	SD	~24 f	1 h food, 24 h wheel	Resilience and susceptibility to ABA: resilient animals have lower alpha 4 GABA receptors in hippocampus
Aravich et al. (1995)	1993	Rats	ABA	SD	80 m	1.5 h food, 22.5 h wheel	Intervention in ABA: 2-deoxy-D-glucose (2DG) injection reduced food intake
Atchley and Eckel (2005)	2005	Rats	ABA	Long Evans	24 f	2 h food, 24 h wheel	Fenfluramine (serotonin agonist) treatment in ABA: increases weight loss
Atchley and Eckel (2006)	2006	Rats	ABA	Long Evans	17 f	2 h food, 24 h wheel	8-OH-DPAT treatment (reduces serotonin) in ABA: prevents weight loss
Avraham et al. (1996)	1996	Mice	Reduced calories	Sabra	103 f	60 and 40% of daily calories	Specific diet: tyrosine high food improved cognitive function
Avraham et al. (2017)	2017	Mice	Reduced time	Sabra	50 f	2.5 h food	Injection treatment: 2-arachidonylglycerol increases food intake
Barbarich-Marsteller et al. (2013a)	2013	Rats	ABA	SD	32 f	1 h food, 24 h wheel	Brain changes in ABA: ABA reduces cell proliferation in hippocampus
Barbarich-Marsteller et al. (2005)	2005	Rats	ABA	Wistar	9 f	40% of baseline food intake, 24 h wheel	Imaging in ABA: first study using micro PET imaging of rats
Barbarich-Marsteller et al. (2013b)	2013	Rats	ABA	SD	80 f	1 h food, 24 h wheel	Susceptibility to ABA: defining vulnerability subtypes
Belmonte et al. (2016)	2016	Mice	ABA	C57BL/6	48 f	3 h food, 24 h wheel	Immune system in ABA: TLR4 upregulated in ABA
Breton et al. (2020)	2019	Mice	ABA	C57BL/6	32 f	3 h food, 24 h wheel	Metabolic changes in ABA: analysis of fecal metabolite changes in ABA
Brown et al. (2008)	2008	Rats	ABA	SD	66 m	1 h food, 24 h wheel	Special diet: high fat diet prevents ABA
Campos et al. (2019)	2019	Rats	Food restriction	Fischer	84 f	40% of control animal food intake	Anxiety and behavior: Estrogen receptor beta activation reverses anxiety like behavior
Carrera et al. (2009)	2009	Rats	ABA	SD	144 (72 m, 72 f)	1.5 h food, 22.5 h wheel	Female rats that had longer times of maternal separation are more resilient to ABA
Casteels et al. (2014)	2014	Rats	ABA	Wistar	80 (23 m, 57 f)	1.5 h food, 24 h wheel	Endocannabinoid system: changes in endocannabinoid transmission in PET imaging
Chen et al. (2018)	2018	Mice	ABA	C57Bl/6J wild-type and α4-subunit of GABA A receptors (α4) KO	90 (35 m, 36 f WT and 9 m KO and 10 f KO)	2 h food, 24 h wheel	Resilience to ABA: female mice with upregulation of alpha4 GABA A receptors were more resilient, but not male
Chen et al. (2017)	2017	Rats	ABA	SD	32 f	1 h food, 24 h wheel	Susceptibility to ABA: NR2A-NMDA receptors correlate with physical activity in ABA
Cerrato et al. (2012)	2012	Rats	ABA	SD	48 f	1.5 h food, 22.5 h wheel	Heat (ambient temperature 32°C) helps rats reverse ABA and maintain body weight
Chowdhury et al. (2013a)	2013	Rats	ABA	SD	16 f	1 h food, 24 h wheel	Brain changes in ABA: apical dendritic branching in dorsal and ventral hippocampal CA1 might explain anxiety
Chowdhury et al. (2014)	2014	Rats	ABA	SD	30 f	1 h food, 24 h wheel	Brain changes in ABA: hippocampal changes depend on whether ABA was started during adolescence or adulthood
Chowdhury et al. (2013b)	2013	Mice	ABA	C57BL/6	23 f	1 h food, 24 h wheel (*n* = 13, only 1 in 5 survived 3 days), 2 h food 24 h wheel (*n* = 10, all 10 survived 3 days)	Resilience to ABA: Mice with hippocampal CA1 pyramidal cells that receive more glutamic contacts are more resilient
Collu et al. (2020)	2020	Rats	ABA	SD	64 f	1.5 h food, 22.5 h wheel	Inflammatory processes: ABA altered central inflammatory pathways
Collu et al. (2019)	2019	Rats	ABA	SD	36 f	1.5 h food, 22.5 h wheel	Hormonal changes: Impaired brain endocannabinoid tone in ABA
Duclos et al. (2005)	2005	Rats	ABA	Fischer 344, Brown Norway and Lewis	72 m	1.5 h food, 22.5 h wheel	Rat strain differences in ABA and HPA axis involvement in running activity
Endou et al. (2001)	2001	Rats	ABA	SD	36 m	1.5 h food, 22.5 h or 24 h wheel	Neurotransmitters: ABA decreased histaminergic neuron system activity
Farinetti et al. (2020)	2019	Rats	ABA	SD	48 (24 m 24 f)	1 h food, 2 h running wheel (just before food)	Maternal separation and ABA: maternally separated female ABA rats were more hyperactive, male not
Filaire et al. (2009)	2009	Rats	ABA	Wistar	56 m	1 h food, 23 h wheel	Lipid peroxidation and antioxidant status in ABA rats
Fraga et al. (2020)	2020	Rats	ABA	SD	74 m	60% of baseline food, 24 h wheel	Heat/increased room temperature is better at preventing ABA than leptin effects
Francois et al. (2015)	2015	Mice	ABA	C57Bl/6	32 m	3 h food, 24 h wheel	Ghrelin in ABA: ABA mice have more preproghrelin mRNA expressing cells in the stomach
Frintrop et al. (2018a)	2018	Rats	Chronic ABA	Wistar	41 f	40% of baseline food intake until 25% body weight loss (acute starvation), then stable weight with adjusted food (chronic starvation), 24 h wheel	Brain changes: reduced astrocyte density might be cause of brain volume reduction in ABA
Frintrop et al. (2019)	2019	rats	Chronic ABA	Wistar	47 f	40% of baseline food intake until 25% body weight loss (acute starvation), then stable weight with adjusted food (chronic starvation), 24 h wheel	Imaging in ABA: longitudinal MRI and post mortem study of brain volume loss in ABA
Frintrop et al. (2018b)	2018	Rats	Chronic ABA	Wistar	53 f	40% of baseline food intake until 25% body weight loss (acute starvation), then stable weight with adjusted food (chronic starvation), 24 h wheel	ABA protocol: development of a more chronic ABA model
Gelegen et al. (2007)	2007	Mice	ABA	C57BL/6J and DBA/2J	C57BL/6J (*n* = 14) and DBA/2J (*n* = 15) all f	2 h food, 24 h wheel	Mice strain differences in susceptibility to ABA
Gelegen et al. (2010)	2010	Mice	ABA	Each strain in the panel has a chromosome pair substituted from the A/J strain on to a host C57BL/6J background	321 f	2 h food, 24 h wheel	Mice strain differences in susceptibility to ABA and chromosomal mapping of susceptibility to ABA (excessive running)
Gelegen et al. (2008)	2008	Mice	ABA	36 C57BL/6J and 21 A/J, some dopamine transporter knockout	57 f	2 h food, 24 h wheel	Neurotransmitters: dopamine receptor D2 expression in the caudate putamen increased in ABA and BDNF expression in the hippocampus reduced
Giles et al. (2016)	2016	Rats	ABA	SD	f	1 h food, 23 h wheel	Refeeding after ABA: ABA rats had more hepatic lipid accumulation
Gilman et al. (2019)	2019	Rats	ABA	SD	73 f/m	1 h food, 24 h wheel	Neurotransmitter: ABA modulates dopamine transporter functional plasticity during adolescence
Gutiérrez et al. (2006)	2006	Rats	ABA	Wistar	32 m	1.5 h food, 22.5 h wheel	High ambient temperature (27–29°C) decreases rate of body weight loss in ABA
Gutierrez et al. (2008)	2008	Rats	ABA	SD	48 m	1.5 h food, 22.5 h wheel	Heat prevents ABA
Gutierrez et al. (2009)	2009	Rats	ABA	SD	24 m	1.5 h food, 22.5 h wheel	Heat prevents ABA and reverses hypothalamic MC4 overexpression in ABA animals
Hancock and Grant (2009)	2009	Rats	ABA	SD	94 (48 m, 46 f) half adolescent and half adult	2 h wheel followed by 1 h food	Maternal separation: maternally separated adolescent ABA rats ran more and ate less
Hao et al. (2001)	2001	Mice	separation induced	Sabra	70 f	Separation into single cage	Tyrosine improves separation induced body weight loss and impairment in cognitive behavior
Hata et al. (2019)	2019	Mice	Stool transplant by AN patient	Germ-free BALB/c	80 f	Food intake *ad libitum*	Intervention: stool transplant of anorexic patient stool leads to anorexia in mice
Hillebrand et al. (2006b)	2006	Rats	ABA	Wistar	29 f	1 h food, 24 h wheel	Neurotransmitter: MSH and Agouti related peptide involvement in ABA
Hillebrand et al. (2005b)	2005	Rats	ABA	Wistar	64 f	1 h food, 24 h wheel	Hormonal treatment: leptin treatment aggravates ABA
Hillebrand et al. (2005c)	2005	Rats	ABA	Wistar	30 f	1 h food, 24 h wheel	Drug treatment in: olanzapine treatment reduced physical activity
Hillebrand et al. (2005a)	2005	Rats	ABA	Wistar	13 f	1 h food, 24 h wheel	Warm plate access reduced running in ABA and weight loss
Hillebrand et al. (2006a)	2006	Rats	ABA	Wistar	30 f	1 h food, 24 h wheel	Treatment with appetite suppressant d-fenfluramine reduced water intake, but not food intake
Ho et al. (2016)	2016	Mice	ABA	Balb/cJ	28 f	6 h food, 24 h wheel	Neurotransmitter: BDNF expression in mesocorticolimbic reward circuit
Hurel et al. (2019)	2019	Mice	ABA	C57BL/6N	32 m/f	50% amount of food, 24 h wheels	Post-weaning isolation trauma in mice
Jean et al. (2012)	2012	Mice	ABA	Male KO1B, KO4 and WT 129/SvPas	40 m	80% amount of food, 24 h wheel	Neurotransmitter: MDMA in mice with ABA
Jean et al. (2017)	2017	Mice	Restraint-stress induced hypophagia and Overexpression of 5-HT4Rs in the mPFC	129SvPas WT, 129SvTer 5-HT4R KO, and WT mice	60 m	Restraint-stress and Overexpression of 5-HT4Rs in the medial prefrontal cortex	5-HT4 receptor expression in the medial prefrontal cortex rescues hypophagia
Jesus et al. (2014)	2014	Mice	ABA	C57Bl/6	40 m	3 h food, 24 h wheel	Intestinal barrier dysfunction in ABA mice
Johansen et al. (2000)	2000	Mice	anx/anx	anx mice	30 m/f	Genetic aberration	anx mouse model: hypothalamic CART anx and serum leptin are reduced in anx mice
Johnson et al. (1996)	1996	Marmot-set prima-tes	Social isolation	*Callithrix jacchus* jacchus	36 m/f	2 weeks complete social isolation from peers	Social isolation leads to body weight loss in small monkeys
L'Huillier et al. (2019)	2019	Mice	ABA	C57BL/6	58 m	3 h food, 24 h wheel	Glutamine restores colonic permeability in ABA
Kim et al. (2017)	2017	Mice	anx/anx	anx/+ mice and anx/anx	111	anx	anx mouse model: tyrosine kinase receptor Tyro3 enhances lifespan and Npy neuron survival in anx mice
Kinzig and Hargrave (2010)	2010	Rats	ABA	Long Evans	39 f	1 h food, 24 h wheel	Behavior: ABA in adolescence increases anxiety in adults
Klenotich et al. (2015)	2015	Mice	ABA	Balb/cJ	98 f	6 h food, 24 h wheel	Drug treatment: amisulpride (D2 antagonism) reduces ABA
Klenotich et al. (2012)	2012	Mice	ABA	Balb/cJ and A/J	102 f	6 h food, 24 h wheel	Drug treatment: olanzapine treatment increases survival in ABA
Koh et al. (2000)	2000	Rats	ABA with alley	SD	24 m	1 h food, 24 h alley or wheel	Circular alley does not work like wheel running activity in ABA
Kumar and Kaur (2013)	2013	Rats	Wistar	Intermittent fasting 24 h on/24 h off	24 m/f	24 h food *ad lib*, 24 h no food, alternating	Intermittent fasting negatively effects on estrous cycle in rats
Legrand et al. (2016)	2016	Mice	C57Bl/6	ABA	59 m	3 h food 24 h wheel	Ghrelin: ghrelin treatment prevents ABA
Lewis and Brett (2010)	2010	Mice	C57/BL6	ABA	112 m	3 h food 21 h wheel	Endocannabinoid system: THC decreased survival in ABA, but increased feeding in survivors
Liang et al. (2011)	2011	Rats	SD	ABA	43 f	1. group 2 h food, 2. group 1 h food, 24 h wheel	Food aversion learning is stronger in rats after recovery of ABA
Lindfors et al. (2015)	2015	mice	anx/anx	anx	47 (21 anx) m/f	anx	anx mouse model: glucose intolerance in anx mice
Lujan et al. (2006)	2006	Rhesus monkey	Reduced food intake		5 f	Dietary restriction in calories	Caloric restriction leads to amenorrhea in rhesus monkeys
Lutter et al. (2017b)	2017	Mice	Original mice were B6/CBA F1 hybrid mice and 87.5% C57BL/6, then heterozygous for HDAC4A778T		64 m/f	HDAC4A778T mutation	Female mice heterozygous for HDAC4A778T display several eating disorder related feeding and behavioral deficits
Madra and Zeltser (2016)	2016	Mice	Val66Met genotype	Genotype, social isolation, juvenile caloric restriction	40 f	20–30% dietary restriction, genetic susceptibility	Female mice with genetic susceptibility to anxiety, decrease food intake on social isolation
Mequinion et al. (2015b)	2015	Mice	C57BL/6J	ABA	82 f	Quantitative food restriction comprising 30%/day for 3 days and then 50%/day until the end of protocol, 24 h wheel	Physical activity in ABA
Mequinion et al. (2017)	2017	Mice	C57BL/6J	Food restriction	24 f	Gradually restricted to 2 h a day	Persistent hypoleptinemia in mice after recovery from decreased body weight
Mercader et al. (2008)	2008	Mice	anx/anx	anx	6 m	anx	anx mouse model: hypothalamus transcriptome profile
Nakahara et al. (2012)	2012	Mice	C57BL/6 J	Valine deficient diet	36 m	Valine deficient diet	Specific diet: valine deficient diet leads to weight loss
Nobis et al. (2018a)	2018	Mice	C57Bl/6	ABA	48f	3 h food, 24 h wheel	Intestinal barrier: analysis of colonic mucosal proteome
Nobis et al. (2018c)	2018	Mice	C57Bl/6	ABA	24 f	3 h food, 24 h wheel	ABA mice have delayed gastric emptying
Nobis et al. (2018b)	2018	Mice	C57Bl/6	ABA	48 f	Progressive limited food access from 6 h/day (day 6) to 3 h/day (day 9), wheel 24 h	ABA mice show changes in proteome, mitochondrial dynamic and signs of autophagy in the hypothalamus
Paulukat et al. (2016)	2016	Rats	Wistar	ABA	47 f	40% food intake, until 20% weight loss, then 2 weeks maintenance	Memory impairment in ABA is associated with decrease in estrogen
Perez-Leighton et al. (2014)	2014	Rats	SD	ABA	57 m/f	1 h food, 24 h wheel	Spontaneous physical activity predicts susceptibility to ABA in rats
Petrovich and Lougee (2011)	2011	Rats	SD	ABA	25 m, 32f	1 h food, 24 h wheel	Prolonged effects of fear induced feeding cessation in females compared to males
Pjetri et al. (2012)	2012	Mice and rats	11 inbred mouse strains (A/J, AKR/J, BALB/cByJ, C3H/HeJ, C57BL/6J, CAST/EiJ, DBA/2J, FVB/NJ, KK/HlJ, NZW/LacJ, WSB/EiJ, and Wistar	ABA	98 mice, 34 rats	2 h food for mice, 1.5 h food for rats, 24 h wheel	Susceptibility to ABA in 11 inbred mouse strains
Reyes-Haro et al. (2015)	2015	Rats	Wistar	Dehydration induced anorexia	27 f	Dehydration-induced anorexia (DIA) group received a 2.5% NaCl solution as their sole drinking liquid with no food restriction; food-restricted group, a positive control to distinguish between starvation and dehydration effects, received tap water *ad libitum* and the same amount of food consumed by the DIA animals	Dehydration induced anorexia reduces astrocyte density
Reyes-Haro et al. (2016)	2016	Rats	Wistar	Dehydration induced anorexia	24 f	Dehydration-induced anorexia (DIA) group received a 2.5% NaCl solution as their sole drinking liquid with no food restriction; food-restricted group, a positive control to distinguish between starvation and dehydration effects, received tap water *ad libitum* and the same amount of food consumed by the DIA animals	Dehydration induced anorexia reduce astrocyte density in the hippocampus
Rieg and Aravich (1994)	1994	Rats	SD	ABA	30 m	1.5 h food, 22.5 wheel	Drug treatment: Clonidine increases feeding in ABA but not body weight
Scharner et al. (2018)	2018	Rats	SD	ABA	24 f	1.5 h food, 24 h wheel	Neurotransmitter: CRF immunoreactive neurons activated in ABA
Scharner et al. (2016)	2016	Rats	SD	ABA	44 f	1.5 h food, 24 h wheel	Brain changes: mapping of ABA brain with cFOS immunohistochemistry
Scharner et al. (2017)	2017	Rats	SD	ABA	24 f	1.5 h food, 24 h wheel	Brain changes: nesfatin-1 immunoreactive neurons in ABA
Scherma et al. (2017)	2017	Rats	SD	ABA	42 f	1.5 h food, 24 h wheel	Cannabinoid receptor agonists in ABA reduce hyperactivity
Schroeder et al. (2018)	2018	Mice	ICR/CD1	ABA	60 m/f	?	Placental miR-340 mediates vulnerability to ABA in mice
Skowron et al. (2018)	2018	Rats	Wistar	ABA	16 f	?	ABA's rats' weight of their uterus decreased and the number of follicles in the ovaries too
van Kuyck et al. (2007)	2007	Rats	Wistar	ABA	19 m	1.5 h food, 24 h wheel	Micro PET study of ABA
Verhagen et al. (2011a)	2011	Rats and mice	Wistar rats, C57Bl/6 ghrelin receptor knockout	ABA	77 rats 24 mice, all f	2 h food, 24 h wheel	Ghrelin levels are highly associated with activity n ABA
Verhagen et al. (2011b)	2011	Rats	Wistar	ABA	64 f	1.5 h food, 24 h wheel	Leptin reduces hyperactivity
Verhagen et al. (2009a)	2009	Rats	Wistar	ABA	56 f	1.5 h food, 24 h wheel	Dopamine antagonism inhibits ABA
Verhagen et al. (2009b)	2009	Rats	Wistar	ABA	24 f	1.5 h food, 24 h wheel	Dopamine in the NAc was increased during feeding in ABA
Verty et al. (2011)	2011	Rats	SD	ABA	28 f	1.5 h food, 24 h wheel	Cannabinoid system: THC reduces weight loss
Wable et al. (2014)	2014	Rats	SD	ABA	8 f	1 h food, 24 h wheel	Neurotransmitter: changes in GABA A receptors in amygdala in ABA
Wable et al. (2015a)	2015	Mice	C57BL6	ABA	23 f	2 h food, 24 h wheel	Exogenous progesterone aggravates running in adolescent female mice
Wable et al. (2015b)	2015	Mice	C57BL6	ABA	36 f	2 h food, 24 h wheel	Anxiety is correlated with running in female mice in ABA
Welch et al. (2019)	2019	Mice	D2-Cre BAC transgenic mice	D2 Receptor overexpression	40 m/f		D2 overexpression leads to increased weight loss during restrictive feeding schedule
Welkenhuysen et al. (2008)	2008	Rats	Wistar	ABA	26 f	1.5 h food, 24 h wheel	Intervention: Electrical stimulation in the lateral hypothalamus in ABA did not have significant effects
Wojciak (2014)	2014	Rats	Wistar	Intermittent fasting	48 f	1 group (half the food intake of control group). 2 group 1 day *ad lib*, 1 day starvation, 3 group 2 day *ad lib*, 2 day starvation, etc 4 days on (*ad lib*), 4 days starvation	Intermittent fasting decreased hemoglobin in rats
Wu et al. (2014)	2014	Rats	Wistar	ABA	56 f	1.5 h food, 24 h wheel	Behavior: Investigations of food anticipatory activity
Zgheib et al. (2014)	2014	Mice	C57Bl/6	Separation-based anorexia	48 f	2 h food, separation	Separation based anorexia

*?, unclear; ad lib, ad libitum; f, female; m, male*.

### Species

Of the 108 included studies, 106 used rodents as their experimental species—of these, 64 studies were performed in rats, 40 in mice and 2 in rats and mice. Two studies were performed in monkeys (marmoset and rhesus monkey, respectively).

### Sex

The majority of studies was performed in experimental animals of female sex. Not all authors gave a reason for their sex choice, but if they did, it was always the much higher prevalence of anorexia nervosa in women compared to men. When male animals were used and an explanation was given, it stated that male animals were chosen because they do not have an estrous cycle that could interfere with the experiments. In rats, 43 studies used females and 13 studies used males. In mice, 21 studies used females and 10 males. One of the two monkey studies used females and the other used males and females. A few rodent studies used animals of both sexes and looked specifically for sex differences (8 studies in rats and 7 in mice).

### Animal Models

Different animal models and experimental protocols were used in the 108 included studies (Table 1). We assessed and divided them into 18 groups. One overall common finding is that most study protocols state that after a 25 or 30% weight loss, animals are taken out of the experiment for ethical reasons. Some studies had additional parameters for termination of the experiment: food intake in rodents below 2 g/24 h or signs of stress.

### Environmental Models

#### Activity-Based Anorexia (ABA)

In total, 81 of the 108 analyzed studies used the ABA model. Therefore, the ABA model is by far the most used animal model to mimic anorexia nervosa. The ABA model is a rodent model that combines food restriction (usually time restricted to a few hours daily) with the possibility for the rodents to run in a running wheel. The combination of these two factors leads, in rodents, to significant weight loss. The extent of the weight loss is mainly dependent on the length of the time period that the animals have access to food. There are two opposing ideas among ABA researchers whether the animals should have constant access (24 h) to the running wheel or whether during the period of feeding the running wheel should be blocked. Usually, studies in which the running wheel gets blocked during the feeding time reason that they do not want to bring the animals in a conflict of whether they prefer running or eating; but in studies that do not block the running wheel, it is often argued that bringing the animals in this conflict is an interesting aspect of this model.

In the ABA studies included in this review, rats and mice were used. Younger animals (adolescents) are more susceptible to developing ABA, but the model works with adult animals as well. Both, in rats and mice, animals usually get a time period in which they get food a*d libitum* but are already in the cage in which they have access to the running wheel. This habituation period usually provides a baseline for running activity and for food intake for each animal. Depending on the experimental protocol, the habituation period lasts between 3 to 10 days with 7 days being a very commonly used time. The period is followed by food restriction. In rats, animals usually have access to food for either 1, 1.5, or 2 h per day. In mice it is more common to have a gradual food restriction from *ad libitum* 24 h access to food to 6 h a day for 3 days for example, and then a further decrease to 3 h a day.

#### ABA With Circular Alley Instead of Wheel

Koh et al. tested whether activity in a flat circular alley also produces the ABA syndrome (Koh et al., 2000). They compared animals that had 24 h access to a wheel with animals that had 24 h access to a circular alley. Both groups had access to food for 1 h per day. The animals with the alley did not develop ABA and in contrast to wheel running, their amount of activity in the alley decreased over days (Koh et al., 2000). The researchers hypothesized that alley activity, in contrast to wheel running, may not be reinforcing and that most likely a physical activity must be reinforcing in order to lead to ABA development (Koh et al., 2000).

#### Chronic ABA in Rats

The ABA model has evolved to be the most commonly used animal model for anorexia nervosa. The chronic ABA model has a changed protocol and tries to address one of the common critique points of the ABA model: that ABA is a very acute model and does not reflect the chronicity of the disease anorexia nervosa. Three studies used the chronic ABA model developed by the research group of Frintrop et al. (2018b). In the chronic ABA model, animals also have access to a running wheel the whole day and receive 40% of their individual baseline food intake (Frintrop et al., 2018b). This regimen is maintained until a 20 to 25% body weight reduction has been reached, which in ABA is the typical period of acute starvation (Frintrop et al., 2018b). Afterwards, rats receive a daily adjusted amount of food to maintain their lower body weight for 14 more days which is the period of chronic starvation (Frintrop et al., 2018b). Female rats experience a complete cessation of the estrous cycle in this model (Frintrop et al., 2018b).

#### Chronic ABA in Mice

Mequinion et al. established a mouse model in C57Bl/6 mice for chronic ABA (Mequinion et al., 2015b). They used a quantitative food restriction comprising 30%/day of baseline food intake for 3 days and then 50%/day of baseline food intake until the end of protocol (Mequinion et al., 2015b). The mice had 24 h access to a wheel. In this protocol, the mice with a running wheel reached a crucial point of body weight loss (especially fat mass) faster than mice with food restriction only (Mequinion et al., 2015b). However, in contrast to the food-restricted control mice, their body weight stabilized, giving rise to a protective effect of moderate, regular physical activity (Mequinion et al., 2015b). The long-term nature of the protocol induced alterations in bone parameters similar to those observed in anorexia patients. Both food-restricted groups differentially adapted their energy metabolism in the short and long term, with less fat oxidation in food-restricted mice with a running wheel and a preferential use of glucose to compensate for the chronic energy imbalance (Mequinion et al., 2015b). Similar to patients with restrictive anorexia nervosa, mice exhibited low leptin levels, high plasma concentrations of corticosterone and ghrelin as well as a disruption of the estrous cycle (Mequinion et al., 2015b).

#### Activity

Adams et al. used a model of voluntary activity in rats that led to food intake suppression (Adams et al., 2009). Rats had 24 h *ad libitum* access to a running wheel which led to an average food intake suppression of 5 g per day (from 30 g to 25 g daily) (Adams et al., 2009).

#### Reduced Calories

Two studies used caloric restriction as a mean to investigate cognitive and behavioral effects of the reduced body weight in animals. The caloric intake was reduced to 60 and 40% of baseline caloric intake in mice in one study (Avraham et al., 1996) and to 40% of control animals' food intake in the second study (Campos et al., 2019). Cognitive function was evaluated using a modified eight-arm maze with rewards. Animals fed to 60% of controls showed improved maze performance while this was significantly impaired in animals on food restriction to 40% (Avraham et al., 1996). However, in these animals, injections of tyrosine restored performance (Avraham et al., 1996). The second study showed that animals with food restriction showed more anxiety- like behavior than controls (Campos et al., 2019).

#### Reduced Calories in Rhesus Monkeys

Lujan et al. investigated in four normal-weight and one obese female rhesus monkeys the relationship between caloric intake and amenorrhea (Lujan et al., 2006). The weight loss required to inhibit ovulation ranged from 2 to 11% in the four normal-weight animals and was achieved with a 23% reduction in dietary intake (Lujan et al., 2006). The animals were provided a healthy diet with low caloric food. From the first day of reduced food intake to first missed ovulation was on average 62 ± 13 days. In the obese monkey only after 10 months of food reduction and a weight loss of 46% body weight lead to inhibition of ovulation (Lujan et al., 2006). The onset of anovulation was not preceded by changes in menstrual cycle length or progesterone secretion (Lujan et al., 2006). When animals were allowed free access to food again, ovulation restarted typically at a body weight close to the animal's weight at the time of the last ovulatory cycle during dietary restriction (Lujan et al., 2006). By contrast, caloric intake at the return of ovulation during realimentation was 28% greater than before amenorrhea (Lujan et al., 2006).

#### Reduced Time of Food Access per Day

Two studies initiated weight loss in mice by reducing their access time to food to 2.5 h per day (Avraham et al., 2017) or a gradual reduction down to 2 h per day (Mequinion et al., 2017).

#### Intermittent Fasting

Two studies used intermittent fasting schedules in rats. Kumar et al. investigated the effects on the HPA axis of an intermittent fasting schedule with 24 h *ad libitum* access to food alternating with 24 h without access to food in female and male rats (Kumar and Kaur, 2013). They observed significant changes in body weight, blood glucose, estrous cyclicity and serum estradiol, testosterone and LH level indicating a negative role of the intermittent fasting regimen on reproduction in these young animals (Kumar and Kaur, 2013).

Wojciak et al. examined the effect of five different feeding regimens on iron and hemoglobin levels in the blood of rats (Wojciak, 2014). They had one group that received 50% of the food intake of a control group. The second group had 1 day *ad libitum* access to food alternating with 1 day of no access to food. The third group had 2 days *ad libitum* access to food alternating with 2 days of no access to food. The fourth group had 3 days *ad libitum* access alternating with 3 days of no access to food. The fifth group had 4 days *ad libitum* access to food and 4 days no access to food (Wojciak, 2014). They found that the longer the starvation the stronger the negative effect on blood concentrations of hemoglobin, hematocrit, RBC, serum ferritin and iron levels in different organs (but even in rats with acute starvation a decrease in these parameters was observable) (Wojciak, 2014).

#### Valine-Deficient Diet

Nakahara et al. fed mice a special diet that was deficient in the essential amino acid valine (Nakahara et al., 2012). The ingestion of this diet results in a significant reduction of food intake and body weight within 24 h, and this phenomenon continues throughout the period over which such a diet is supplied (Nakahara et al., 2012). Nakahara et al. investigated the mechanisms that lead to this weight loss and found that the expression of somatostatin mRNA is increased in the hypothalamus in the mice that received a Valine-deficient diet (Nakahara et al., 2012). They reported, too, that when somatostatin was administered intracerebroventricularly (icv) to normal weight animals that were fed a control diet, their 24 h food intake decreased significantly (Nakahara et al., 2012).

#### Dehydration-Induced Anorexia

Reyes-Haro et al. used dehydration-induced anorexia to examine specific effects of this model on the brain in Wistar rats (Reyes-Haro et al., 2015, 2016). The dehydration-induced anorexia group received a 2.5% NaCl solution as their sole drinking liquid with no food restriction (Reyes-Haro et al., 2015, 2016). Additionally, they had a food-restricted group which served as positive control to distinguish between starvation and dehydration effects. The food-restricted group received tap water *ad libitum* and the same amount of food consumed by the dehydration-induced anorexia animals. The authors described that in dehydration-induced anorexia rats the astrocyte density was significantly reduced (~34%) in the body of the corpus callosum (but no changes in the genu and the splenium callosum) (Reyes-Haro et al., 2015) and the glia cell density was about 20% reduced in all regions of the hippocampus, except in CA1 (Reyes-Haro et al., 2016).

#### Separation-Induced Anorexia in Mice

Hao et al. separated mice into single cages (Hao et al., 2001) which led to self-induced weight loss caused by separation stress. Separation significantly decreased body weight in mice (Hao et al., 2001). They showed in their study, that tyrosine-rich food in mice with separation-induced weight loss and decreased cognitive function, restored their cognitive function and restored their separation-induced low dopamine levels (Hao et al., 2001).

#### Separation-Induced Anorexia in Monkeys

Johnson et al. investigated body weight loss in marmoset primates. The animals were in complete social isolation from peers for 2 weeks (Johnson et al., 1996). All animals (male and female) lost close to 10% (around 25 g) of their body weight after 2 weeks of social isolation (Johnson et al., 1996).

#### Separation Combined With Reduced Time Access to Food

Zgheib et al. combined a reduced access time to food of 2 h per day and separation into single cages in mice (Zgheib et al., 2014). The animals displayed marked alterations in body weight, fat mass, lean mass, bone mass acquisition, reproductive function, GH/IGF-1 axis and circulating leptin levels (Zgheib et al., 2014). All these alterations were corrected during the recovery phase, except for the hypoleptinemia that persisted despite full recovery of fat mass (Zgheib et al., 2014).

### Procedure-Induced Models

#### Stool Transplantation of Patients With Anorexia Nervosa to Mice

Hata et al. investigated the effects of stool transplantation from human patients with restrictive anorexia nervosa in germ-free mice (Hata et al., 2019). The female offspring of the anorexia patient-stool transplanted mice showed a decrease in body weight gain, concomitant with reduced food intake compared to the female offspring of mice that received stool from healthy individuals (Hata et al., 2019). Food efficiency ratio (body weight gain/food intake) was also significantly lower in the female offspring of anorexia patient-stool transplanted mice than in the female offspring of healthy individuals-stool transplanted mice, suggesting that decreased appetite as well as the capacity to convert ingested food to unit of body substance may contribute to poor weight gain (Hata et al., 2019).

### Genetic Models

#### Anx/anx

Four of the 108 included articles used the anx/anx genetic mouse model. The anorexia *(anx)* mutation is an autosomal recessive mutation detected in 1984 by Maltais et al. that causes starvation in mice. *anx/anx* mice appear normal at birth, but develop growth failure and low body weight, even appearing emaciated, as well as neurological motor disturbances (e.g., head weaving (head moving up and down), gait abnormalities and hyperactivity) (Maltais et al., 1984). Usually, they die early, between the age of 3–5 weeks, due to severe malnutrition. The amount of milk consumed by *anx*/*anx* mice is significantly lower than for littermate controls and leads to a caloric deficit. Researchers have made differing statements about how similar this model is to anorexia nervosa, but many stated that this mutation might play an important role as a model system for the study of basic feeding drive (Maltais et al., 1984).

Mercader et al. performed an expression profiling in the hypothalamus of the *anx/anx* mice (Mercader et al., 2008). Their results show enrichment in deregulated genes involved in cell death, cell morphology and cancer, as well as an alteration of several signaling circuits involved in energy balance including neuropeptide Y and melanocortin signaling (Mercader et al., 2008).

Johansen et al. showed that in mice with the genetic aberration *anx/anx* levels of CART mRNA and peptide-immunoreactive cell bodies and fibers in the arcuate nucleus were decreased and additionally a lower number of detectable CART-expressing cells in the dorsomedial hypothalamic nucleus/lateral hypothalamic area was observed (Johansen et al., 2000). Moreover, serum leptin levels were significantly lower in *anx/anx* mice compared to normal littermates (Johansen et al., 2000).

Kim et al. identified a mutation (C19T) that converts arginine to tryptophan (R7W) in the TYRO3 protein tyrosine kinase 3 (*Tyro3*) gene, which resides within the *anx* critical interval, likely contributing to the severity of the *anx* phenotype (Kim et al., 2017). *Tyro3* is expressed in the hypothalamus and other brain regions affected by the *anx* mutation, and its mRNA localization appeared abnormal in *anx/anx* brains by postnatal day 19 (Kim et al., 2017). The presence of wild-type *Tyro3* transgenes, but not an *R7W-Tyro3* transgene, doubled the weight and lifespans of *anx/anx* mice and near-normal numbers of hypothalamic neuropeptide Y expressing neurons were present in *Tyro3*-transgenic *anx/anx* mice (hypothalamic neuropeptide Y expressing neurons are reduced in anx/anx mice) (Kim et al., 2017). Further analyses indicated that the C19T *Tyro3* mutation is present in a few other mouse strains, and hence is not the causative *anx* mutation, but rather an *anx* modifier (Kim et al., 2017).

Lindfors et al. investigated glucose tolerance in *anx*/*anx* mice (Lindfors et al., 2015). *anx/anx* exhibit marked glucose intolerance associated with reduced insulin release following an intraperitoneal (ip) injection of glucose (Lindfors et al., 2015). In contrast, insulin release from isolated *anx*/*anx* islets is increased after stimulation with glucose or KCl (Lindfors et al., 2015). In addition, they show elevated concentrations of free fatty acids in *anx*/*anx* serum and increased macrophage infiltration (indicative of inflammation) in *anx*/*anx* islets (Lindfors et al., 2015).

#### Heterozygous for HDAC4A778T

Lutter et al. found that a rare missense mutation in the gene for the transcriptional repressor histone deacetylase 4 (HDAC4) is associated with the risk of developing an eating disorder in humans (Lutter et al., 2017a). To investigate further the biological consequences of this missense mutation, the authors created transgenic mice carrying this mutation by introducing the alanine to threonine mutation at position 778 of mouse Hdac4 (corresponding to position 786 of the human protein) (Lutter et al., 2017b). Female mice heterozygous for HDAC4A778T displayed several eating disorder-related feeding and behavioral deficits depending on housing condition, whereas male mice did not show any behavioral differences (Lutter et al., 2017b). Individually housed HDAC4A778T female mice exhibited reduced effortful responding for high-fat diet and compulsive grooming, whereas group-housed female mice displayed increased weight gain on a high-fat diet, reduced behavioral despair and increased anxiety-like behavior (Lutter et al., 2017b).

#### D2-Cre BAC Transgenic Mice—Overexpression of Dopamine 2 Receptors on Nucleus Accumbens Core Neurons

Welch et al. investigated mice that overexpressed dopamine-2 receptors on nucleus accumbens core (D2R-OENA mice) neurons that endogenously express D2 receptors, and tested mice of both sexes in the open field test, ABA paradigm and the ip glucose tolerance test (Welch et al., 2019). D2R-OENAc did not alter baseline body weight but increased locomotor activity in the open field across both sexes. During constant access to food and running wheels, D2R-OENAc mice of both sexes increased food intake and ran more than controls. However, when food was available only 7 h a day, only female D2R-OENAc mice rapidly lost 25% of their initial body weight, reduced food intake, and substantially increased wheel running (Welch et al., 2019). Surprisingly, female D2R-OENAc mice also rapidly lost 25% of their initial body weight during scheduled fasting without wheel access and showed no changes in food intake. In contrast, male D2R-OENAc mice maintained body weight during scheduled fasting (Welch et al., 2019). D2R-OENAc mice of both sexes also showed glucose intolerance in the IGTT. The findings implicate that the overexpression of D2 receptors in the nucleus accumbens core neurons alters glucose metabolism in both sexes but drives robust weight loss only in females during scheduled fasting (Welch et al., 2019).

#### Overexpression of 5-HT4Rs in the Medial Prefrontal Cortex Neurons + Restraint Stress

Restraint-stress can induce transient hypophagia in mice. In this study by Jean et al., they examined the effects of restraint stress on transgenic mice that overexpressed 5-HT4 receptors in the medial prefrontal cortex (Jean et al., 2017) which is involved in goal-directed behavior (decision making). They showed that mice with this overexpression displayed restraint stress-induced hypophagia that was more persistent than in wild type mice (Jean et al., 2017).

#### Val66Met Genotype + Social Isolation + Juvenile Caloric Restriction

In this study Madra et al. examined mice that were genetically more susceptible to anxiety (Madra and Zeltser, 2016). Female mice with the *hBDNF-*Val66Met allele were exposed to social isolation stress during adolescence and a restricted caloric intake by 20–30% for 11 days (Madra and Zeltser, 2016). Approximately 40% of the female *hBDNF*-Val66Met carriers exposed to early social isolation stress and caloric reduction during adolescence exhibited severe self-imposed dietary restriction, sometimes to the point of death (Madra and Zeltser, 2016).

### Important Findings in ABA (Table 1)

#### Sex Differences

Achamrah et al. reported greater susceptibility of male mice to develop ABA leading to a higher mortality rate in male mice and slightly different physical activity patterns (Achamrah et al., 2016a).

ABA rats of both sexes display hyperactive behavior associated with reduced anxiety-like behavior when compared to controls in tests like open field and elevated plus maze (Hancock and Grant, 2009; Farinetti et al., 2020). Farinetti et al. further investigated this phenomenon and found a sexually dimorphic effect of early maternal separation in ABA rats: female rats exposed to maternal separation+ ABA were even more hyperactive with further diminished anxiety-related behaviors compared to females of ABA group, while in male rats maternal separation did not exert an additional effect to their behavior (Farinetti et al., 2020). Hancock et al. examined sex differences in early separation and ABA as well and found age-specific sex differences: compared to handled females, maternally separated females demonstrated greater increases in wheel running and a more pronounced running-induced suppression of food intake during adolescence, but not in adulthood (Hancock and Grant, 2009). In contrast, it was only in adulthood that wheel running produced more prolonged anorexic effects in maternally separated males than in handled males (Hancock and Grant, 2009).

Petrovich et al. tested the phenomenon of fear induced-food cessation in ABA rats (by combining electro shocks to one foot with a tone and then playing just that tone during food intake) (Petrovich and Lougee, 2011). They found that female rats showed sustained fear-cue induced feeding inhibition compared to males during the extinction (the period in which the rats “unlearn” that the tone is associated with pain (Petrovich and Lougee, 2011).

One study by Skowron et al. looked specifically at the effect of ABA on female reproductive organs during the cessation of the estrous cycle (Skowron et al., 2018). They reported that in the ABA group the weight of the uteri and the number of follicles in the ovaries decreased significantly (Skowron et al., 2018).

#### Rodent Strain Differences

The most commonly used rat strains were Sprague-Dawley or Wistar rats, which are known to be rather physically active. However, a few studies used other rat strains like Long Evans, which is known to be less active. All rat strains included in this review developed ABA.

Most studies involved Wistar and Sprague Dawley strains. Duclos et al. tested Brown Norway, Lewis and Fischer rats in the ABA model (Duclos et al., 2005). They found that Brown Norway and Lewis rats lost 25% of body weight faster than Fischer rats (Duclos et al., 2005). Additionally, they tested daily the prefeeding corticosterone levels in the blood of the rats which were increased in the two more susceptible rat strains under ABA conditions, while no rise was observed in Fischer rats (Duclos et al., 2005).

In mice by far most studies were performed on C57BL/6J mice (Gelegen et al., 2007). Gelegen et al. tested the differences between C57BL/6J and DBA/2J inbred mouse strains because they have been previously reported as having low and high anxiety, respectively (Gelegen et al., 2007). C57BL/6J mice during ABA reduced their wheel activity, in contrast to DBA/2J mice which exhibited increased physical activity (Gelegen et al., 2007). Food restriction induced hypoleptinemia in both strains, but the decline in plasma leptin was stronger in DBA/2J mice and correlated with increased activity only in that strain (Gelegen et al., 2007). In a further study they investigated a panel of mouse chromosome substitution strains derived from C57BL/6J and A/J strains and their reaction to ABA (Gelegen et al., 2010). They showed that A/J chromosomes 4, 12, and 13 contribute to the development of excessive running wheel activity in response to daily restricted feeding and hence, accelerated weight loss (Gelegen et al., 2010). Gelegen et al. mentioned that regions on mouse chromosomes 4, 12, and 13 display homology with regions on human chromosomes linked with anxiety and obsessionality in anorexia cohorts (Gelegen et al., 2010).

Pjetri et al. tested the ABA model on 11 different strains of mice (A/J, AKR/J, BALB/cByJ, C3H/HeJ, C57BL/6J, CAST/EiJ, DBA/2J, FVB/NJ, KK/HlJ, NZW/LacJ, and WSB/EiJ) and Wistar rats and found that baseline wheel running activity levels preceding the scheduled food restriction phase strongly predicted activity-based anorexia susceptibility compared to other baseline parameters (Pjetri et al., 2012).

#### Vulnerability/Susceptibility to ABA

Perez-Leighton et al. reported that baseline spontaneous physical activity before ABA (voluntary in a cage without a running wheel) can predict the baseline running activity and the probability of high weight loss in rats (Perez-Leighton et al., 2014).

The research group of Chen et al. examined gender-specific vulnerability to ABA in mice (Chen et al., 2018). ABA led to an overall suppression of wheel running (compared to baseline) but there was a sex-specific effect: suppression of wheel running occurred during the food-anticipatory hours in males, while in females suppression was observed during food-access hours. Correspondingly, only females adaptively increased food intake (Chen et al., 2018). Another study reported that rats with the highest body weight loss had the lowest level of food-anticipatory activity (running in the wheel during the time period of 4 h before feeding) and that postprandial activities are more directly predictive of weight loss (Wu et al., 2014). Barbarich-Marsteller et al. tried to identify vulnerable subtypes to ABA and found that rats with maximal hyperactivity, minimal food intake, and the shortest time to experimental exit were most vulnerable, while those with minimal activity and the longest time to experimental exit were more resistant (Barbarich-Marsteller et al., 2013b).

As infant/adolescent trauma is a risk factor for the development of anorexia nervosa, the study by Hurel et al. analyzed the impact of post-weaning isolation on body weight and wheel-running performance in female mice exposed to an ABA protocol (Hurel et al., 2019). Post-weaning isolation amplified ABA-elicited body weight reduction and stimulated wheel-running activities in anticipation of feeding in female mice compared to controls (Hurel et al., 2019).

Schroeder et al. found that by screening placental microRNA expression of naive and prenatally stressed fetuses and assessing vulnerability to ABA that miR-340 might be a sexually dimorphic regulator involved in prenatal programming of ABA (Schroeder et al., 2018). Prenatal stress caused hypermethylation of placental miR-340, which is associated with reduced miR-340 expression and increased protein levels of several target transcripts linked to the expression of several nutrient transporters both in mice and human placentas (Schroeder et al., 2018).

Carrera et al. showed that female rats that were exposed to longer times of maternal separation (180 min) during their first 20 days of their life were more resilient to ABA (Carrera et al., 2009). Interestingly, they did not see this effect of longer survival times in male rats, neither in rats that experienced only short times of maternal separation (15 min) (Carrera et al., 2009).

#### Effects of Different Diets

Brown et al. examined the effect of a high-fat diet in ABA rats (1 h food access/day, 24 h running wheel) (Brown et al., 2008). Access to the sweet high-fat chow both reversed and prevented the weight loss typical for activity-based anorexia (Brown et al., 2008). Vegetable fat reduced body weight loss, but to a lesser degree than the sweet high-fat diet (Brown et al., 2008). In contrast, addition of saccharin or sucrose solutions to the standard lab chow diet had no effect (Brown et al., 2008).

Giles et al. found that weight restoration on a high carbohydrate refeeding diet promotes rapid weight regain in ABA compared to rats that were food restricted without a running wheel (Giles et al., 2016). Further, they reported that after refeeding ABA rats had higher hepatic lipid accumulation compared to food restricted rats which had more lipid accumulation in visceral adipose tissue despite maintaining the same total body weight in both groups (Giles et al., 2016).

#### Neurocognitive and Behavioral Changes

Some studies show that food-restricted animals display more anxiety behavior (Campos et al., 2019), while others show that they have less anxiety behavior (Wable et al., 2015b) giving rise to other contributing factors. Campos et al. described that estrogen receptor beta activation within the dorsal raphe nucleus reversed anxiety-like behavior induced by food restriction in rats (Campos et al., 2019). This points into the direction that decreased estrogen levels in food-restricted animals lead to anxiety behavior (Campos et al., 2019). Another study reported that food restriction, with or without exercise, reduced anxiety as measured by the proportion of entries into the open arms of the elevated plus maze (Wable et al., 2015b). Moreover, the authors found a correlation that individual ABA animals with less entries (more anxious) displayed more running behavior in the wheel (Wable et al., 2015b).

In Hata et al.'s study of stool transplantation of patients with anorexia nervosa in germ-free mice both anxiety-related behavior measured by open-field tests and compulsive behavior measured by a marble-burying test were increased only in female offspring of mice that received the human anorexia nervosa stool transplantation, but not in the female offspring of the healthy human stool-transplanted mice (Hata et al., 2019).

Kinzig et al. wanted to investigate whether experience with ABA produced enduring effects on brain and behavior (Kinzig and Hargrave, 2010). They tested adult female rats that had experienced ABA during adolescence for anxiety-like behavior and also showed in elevated plus maze and open field test increased anxiety behavior compared to adult rats with only food restriction experience in adolescence (Kinzig and Hargrave, 2010). Lastly, in chronic ABA, starvation disrupted menstrual cycle and impaired memory function (object recognition memory) which became statistically significant in the chronic state compared to control rats (Paulukat et al., 2016). 17 β-estradiol level reduction correlated with the loss of memory in the chronic condition (Paulukat et al., 2016) suggesting a role of estrogens in cognitive functions as well.

#### Neuroendocrine and Cerebral Changes

Aoki et al. showed that female ABA rats exhibit a rise of α4 and δ subunits of α4βδ GABA receptors at puberty onset compared to control animals (Aoki et al., 2012) and that animals that do not develop ABA have lower α4 GABA receptors in CA1 hippocampus than rats that develop ABA (Aoki et al., 2014). They demonstrated that exogenous progesterone exacerbates the running response of adolescent female mice to repeated food restriction stress by increasing alpha4-GABA A receptor expression on hippocampal CA1 pyramid neurons (Wable et al., 2015a). The same research group examined effects of ABA on GABA receptors in the amygdala, too, and found that in ABA mice excitatory synapses on dendritic shafts of the caudal basal amygdala exhibit elevated levels of GABA A receptor α4 subunit (Wable et al., 2014).

The group around Chowdhury examined the effects of ABA on cells in the hippocampus. They showed that cell proliferation was acutely reduced in the hippocampus after 3 days of ABA and that this effect was mainly on gliogenesis and not on neurogenesis (Barbarich-Marsteller et al., 2013a). In the dorsal hippocampus, which preferentially mediates spatial learning and cognition, cells of ABA animals had less total dendritic length and fewer dendritic branches in stratum radiatum than in controls (Chowdhury et al., 2013a). In the ventral hippocampus, which preferentially mediates anxiety, ABA evoked more branching in stratum radiatum than in control animals (Chowdhury et al., 2013a). The authors state that these results may indicate that ABA elicits pathway-specific changes in the hippocampus that may explain the increased anxiety and reduced behavioral flexibility observed in ABA (Chowdhury et al., 2013a). In another study they reported that ABA during adolescence disrupts normal development of the CA1 pyramidal cells in the ventral hippocampus (increased branching), which did not occur in ABA during adulthood suggesting an age-dependent effect on structural plasticity (Chowdhury et al., 2014). They reported, too, that hippocampal CA1 pyramidal cells of ABA-resilient mice receive enhanced glutamic acid decarboxylase contacts compared to the pyramidal cells of ABA-susceptible mice (Chowdhury et al., 2013b). Furthermore, NR2A- and NR2B-NMDA receptors and drebrin within postsynaptic spines of the hippocampus correlated with hunger-evoked exercise and a rat's individual ABA severity (Chen et al., 2017).

Two studies in chronic ABA rats observed the volumes of the cerebral cortex and corpus callosum to be significantly reduced compared to controls by 6 and 9%, respectively (Frintrop et al., 2018a). The number of GFAP-positive astrocytes in these regions decreased, but no changes in neurons were observed (Frintrop et al., 2018a). Furthermore, mean astrocytic GFAP mRNA expression was similarly reduced in ABA animals, as was the mean cell proliferation rate, whereas the mean apoptosis rate did not increase (Frintrop et al., 2019). Longitudinal animal MRI confirmed a reduction in the mean brain volumes of ABA animals compared to controls (Frintrop et al., 2019). After refeeding, the starvation-induced effects were almost completely reversed (Frintrop et al., 2019).

Nobis et al. examined the hypothalamus of ABA mice with proteomic analysis and found changes in the proteome, mitochondrial signaling (increase in fission, no change in fusion) and signs of autophagy (increased dynamin-1, and LC3II/LC3I ratio) (Nobis et al., 2018b) which shows adaptation of the hypothalamic protein synthesis to ABA.

Barbarich-Marsteller et al. performed the first study with micro PET imaging in ABA in 2005 and found increased intake of 18-fluorodeoxyglucose (FDG) in the cerebellum, and decreased in the hippocampus and striatum compared to control animals (Barbarich-Marsteller et al., 2005). In a study from 2007 increased uptake was once again found in the cerebellum and additionally in the mediodorsal thalamus and the ventral pontine nuclei, while decreased uptake was seen in the striatum again and in the left rhinal and bilateral insular cortex (van Kuyck et al., 2007).

In a PET imaging of the type 1 cannabinoid receptor in ABA animals, widespread transient disturbance of the endocannabinoid transmission was shown compared to control animals (Casteels et al., 2014). Another study indicated that cannabinoid receptor 1 density was decreased in the dentate gyrus of the hippocampus and in the lateral hypothalamus (Collu et al., 2019). After recovery, the density was partially normalized in some areas (Collu et al., 2019).

Endou et al. reported that ABA in rats decreased the activity of the histaminergic neuron system and intraventricular administration of histamine significantly reduced the hyperactivity caused by ABA (Endou et al., 2001).

Brain-derived neurotrophic factor (BDNF), an activity-dependent modulator of neuronal plasticity, is reduced in the serum of AN patients, and is a known regulator of feeding and weight maintenance (Ho et al., 2016). Gelegen et al. described reduced BDNF expression in the hippocampus of ABA mice (Gelegen et al., 2008). Ho et al. examined the effects of scheduled feeding and running wheel access on the expression of BDNF transcripts within the mesocorticolimbic pathway (Ho et al., 2016). Conversely, they found that scheduled feeding increased the levels of BDNF mRNA in the hippocampus and decreased BDNF mRNA levels in the medial prefrontal cortex (Ho et al., 2016). In addition, wheel running increased BDNF mRNA expression in the ventral tegmental area (Ho et al., 2016).

Gelegen et al. described increased dopamine receptor D2 expression in the caudate putamen of ABA mice (Gelegen et al., 2008). Gilman et al. studied dopamine transporters in ABA and suggest that ABA modulates dopamine transporter functional plasticity during adolescence in a sex-dependent and age-specific manner (Gilman et al., 2019).

Activation of the melanocortin system leads to hypophagia and increased energy expenditure in *ad libitum* fed rats (Hillebrand et al., 2006b). Pro-opiomelanocortin (POMC) gene expression is normally decreased during negative energy balance (Hillebrand et al., 2006b). Hillebrand et al. reported a transient up-regulation of POMC mRNA levels in the arcuate nucleus during the development of ABA, giving rise to a hyperactive melanocortin system (Hillebrand et al., 2006b). However, ABA development was not influenced by treating ABA rats with the competitive melanocortin antagonist SHU9119 (Adan et al., 2011). Instead, treatment with the inverse agonist Agouti related peptide (AgRP) did ameliorate signs of ABA (Adan et al., 2011) pointing toward constitutive signaling of the receptor.

Jean et al. investigated the addictive facet of anorexia by studying the nucleus accumbens (NAc) (Jean et al., 2012). They found that nucleus accumbens 5-HT_4_ receptor overexpression upregulated NAc-cocaine and amphetamine related transcript (CART) and provoked anorexia and hyperactivity (Jean et al., 2012). NAc-5-HT_4_ knockdown or blockade reduced ecstasy-induced hyperactivity (Jean et al., 2012). Finally, NAc-CART knockdown suppressed hyperactivity upon stimulation of the NAc-5-HT_4_ (Jean et al., 2012).

Verhagen et al. examined the dopamine and serotonin levels in the NAc upon development of ABA (Verhagen et al., 2009b). Surprisingly, the release of dopamine and serotonin in the NAc were not increased during wheel running in the time period just before scheduled feeding time (Verhagen et al., 2009b). Dopamine release in the NAc was increased while eating in ABA rats (Verhagen et al., 2009b). During ABA, levels of serotonin were low and circadian activity was blunted (Verhagen et al., 2009b).

In an investigation of c-Fos expression in the brain of ABA rats, our research group found neuronal activation in several brain nuclei such as the supraoptic nucleus, arcuate nucleus, locus coeruleus and nucleus of the solitary tract of ABA compared to *ad libitum* fed rats, indicating neuronal activation in brain areas involved in the regulation of several functions such as motor activity, stress response, food intake and thermogenesis (Scharner et al., 2016).

Our research group also examined CRF immunohistochemistry in brains of ABA rats (Scharner et al., 2018). ABA increased the number of c-Fos/CRF double labeled neurons in the paraventricular nucleus and the dorsomedial hypothalamic nucleus compared to *ad libitum* fed animals but not to restricted fed rats, pointing toward brain CRF playing a role in the development and maintenance of ABA possibly by activation of nuclei involved in food intake, thermogenesis and circadian rhythms regulation (Scharner et al., 2018).

In another study, we tried to further determine the role of the anorexigenic hormone nesfatin-1 in ABA (Scharner et al., 2017). ABA increased the number of nesfatin-1 immunopositive neurons in the paraventricular nucleus, arcuate nucleus, dorsomedial hypothalamic nucleus, locus coeruleus and in the rostral part of the nucleus of the solitary tract compared to *ad libitum* fed rats but not to restricted fed rats (Scharner et al., 2017). Moreover, we observed significantly more c-Fos and nesfatin-1 double-labeled cells in ABA rats compared to all control groups in the supraoptic nucleus and compared to *ad libitum* fed animals in the paraventricular nucleus, arcuate nucleus, dorsomedial hypothalamic nucleus, dorsal raphe nucleus and the rostral raphe pallidus (Scharner et al., 2017). These results indicate that the observed changes of brain nesfatin-1 might play a role in the pathophysiology and symptomatology under conditions of ABA, since nesfatin-1 plays a role in the inhibition of food intake and the response to stress (Scharner et al., 2017).

Induction of and recovery from ABA altered central COX, LOX and CYP pathways in rats (Collu et al., 2020). Arachidonic acid and arachidonic acid derived eicosanoids levels were altered in corticolimbic brain areas of female rats displaying an ABA phenotype (Collu et al., 2020). mRNA expression of PLA2, ALOX-5 and ALOX-15 enzymes was altered in the nucleus accumbens and caudate putamen regions in ABA rats (Collu et al., 2020).

#### Metabolic System

Filaire et al. showed that food-restricted rats had higher plasma antioxidant concentrations and higher alpha-tocopherol concentrations in the liver when compared to animals fed *ad libitum* (Filaire et al., 2009). They also showed that food restriction coupled to wheel running decreased antioxidant parameters in liver, and plasmatic lipid peroxidation parameters and increased antioxidant plasma concentrations when compared to the *ad libitum* sedentary situation (Filaire et al., 2009). Thus, ABA might have a different effect on antioxidant parameters than food restriction without physical activity, but further research on the different effects and pathways is necessary.

### Gut-Brain Axis

#### Intestinal Barrier

Intestinal barrier alterations in ABA were reported by Jesus et al. Colonic histology showed decreased thickness of the muscularis layer in ABA and colonic permeability was increased in ABA compared to control animals, while jejunal permeability was not affected (Jesus et al., 2014). ABA mice exhibited increased paracellular permeability and reduced protein synthesis in the colonic mucosa (L'Huillier et al., 2019). Oral glutamine supplementation restored colonic paracellular permeability and protein synthesis and increased the mucin-2 mRNA level without affecting body weight (L'Huillier et al., 2019). Colonic mucosal proteome is altered during ABA suggesting a downregulation of energy metabolism (Nobis et al., 2018a). A decrease of protein synthesis and an activation of autophagy were also observed to be mediated by mTOR pathway (Nobis et al., 2018a).

#### Fecal Metabolites

Physical activity altered the abundance of 14 fecal metabolites involved in gut microbial metabolism and proteolysis (Breton et al., 2020). Food restriction only and ABA both disrupted a wide range of metabolic pathways including gut microbial metabolism, proteolysis and fatty acid breakdown (24 urinary and 6 plasma metabolites), but there were no differences between food restricted or ABA animals (Breton et al., 2020) giving rise to food restriction driving the changes in microbiota.

#### Intestinal Inflammatory Status

ABA seems to affect intestinal inflammatory status and the hypothalamic response (Belmonte et al., 2016). Belmonte et al. found that TLR4 was upregulated both on colonic epithelial cells and intestinal macrophages, leading to elevated downstream mucosal cytokine production (Belmonte et al., 2016). Paradoxically, TLR4-deficient mice exhibited greater vulnerability to ABA with increased mortality rate, suggesting a major contribution of TLR4-mediated responses during ABA-induced weight loss (Belmonte et al., 2016).

#### Gastric Emptying

Both food restricted and ABA mice exhibited a delayed gastric emptying compared with controls (Nobis et al., 2018c). ABA mice specifically exhibited an increased rate of gastric oxidized proteins (Nobis et al., 2018c).

#### Ghrelin

Preproghrelin mRNA expressing cells were studied by *in situ* hybridization in mice (Francois et al., 2015). ABA increased their number in the stomach proportionally to body weight loss (Francois et al., 2015). Verhagen et al. described that plasma ghrelin levels were highly associated with food anticipatory behavior, measured by running wheel activity in rats (Verhagen et al., 2011a). Furthermore, they showed that ghrelin receptor (GHS-R1A) knockout mice do not anticipate food when exposed to the ABA model, unlike their wild type littermate controls (Verhagen et al., 2011a). Likewise, food anticipatory activity in the ABA model was suppressed by a GHS-R1A antagonist administered either by acute central (icv) injection in rats or by chronic peripheral treatment in mice (Verhagen et al., 2011a). The GHS-R1A antagonist treatment did not alter food intake in any of these models (Verhagen et al., 2011a). Legrand et al. examined the effects of a single daily intraperitoneal injection of ghrelin together from obese or lean mice before access to food in ABA mice (Legrand et al., 2016). They found that ghrelin from obese, but not lean mice, prevented the hyperactivity typical in ABA, however, they were not able to diminish body weight loss (Legrand et al., 2016).

#### Leptin

Hillebrand et al. examined the effects of chronic leptin treatment (icv, 4 μg/day over 5 days) (Hillebrand et al., 2005b). Leptin treatment decreased running wheel activity in ABA rats and reduced food intake as well as increased energy expenditure by thermogenesis in ABA rats (Hillebrand et al., 2005b). Altogether, this resulted in a stronger negative energy balance and body weight loss in leptin-treated ABA rats (Hillebrand et al., 2005b). Verhagen et al. showed, too, that icv leptin injections and local injections of leptin into the ventral tegmental area suppress running wheel activity (Verhagen et al., 2011b) pointing toward an important role of leptin/hypoleptinemia in physical (hyper)activity.

### Interventions

#### Drug Treatment

Aravic et al. showed that 2-deoxy-D-glucose (2DG) injections paradoxically reduced food intake in ABA (as also seen in human anorexia nervosa where it is associated with decreased subjective hunger ratings) (Aravich et al., 1995). Avraham et al. examined the effects of intraperitoneal 2-arachidonoylglycerol injections in restricted fed mice (Avraham et al., 2017). 2-Arachidonoylglycerol administered 10 min before food intake increased food intake and improved cognition by elevating norepinephrine and L-DOPA in the hippocampus (Avraham et al., 2017).

#### Deep Brain Stimulation

In an experiment of electrical stimulation in the lateral hypothalamus in ABA rats, Welkenhuysen et al. could not show any differences between the ABA animals receiving the stimulation and control animals (Welkenhuysen et al., 2008).

#### Temperature Increase in the Experimental Room

Gutierrez et al. examined the effects of increased ambient temperature in the experimental room on the development of ABA since ABA often leads to hypothermia (Gutiérrez et al., 2006; Gutierrez et al., 2008; Cerrato et al., 2012). They found that increased ambient temperature reduced running rates and led to weight gain in female and male ABA rats (Gutiérrez et al., 2006; Gutierrez et al., 2008; Cerrato et al., 2012). The effect of increasing ambient temperature on food intake in food restricted rats was dependent on whether they had access to a running wheel (ABA) or not (Gutierrez et al., 2008). In their 2006 study they showed that male rats had less weight loss when kept at high ambient temperatures (27–29°C), but even less if their running wheel access was additionally reduced to only 3 h a day (compared to 22.5 h in their normal ABA protocol) (Gutiérrez et al., 2006). In their 2008 study they showed that although warming reduced food intake in the food restricted sedentary rats their body weight remained stable, whereas in ABA rats increased ambient temperature did not reduce food intake and weight gain gradually rose (Gutierrez et al., 2008). Female sedentary food-restricted rats at 32°C ambient temperature were able to maintain the same body weight as the sedentary food-restricted rats at 21°C ambient temperature, but ate 20% less food (Cerrato et al., 2012). Moreover, hypothalamic melanocortin 4 receptors were increased in ABA rats, but reduced in ABA rats at high ambient temperature (32°C) (Gutierrez et al., 2009). Fraga et al. showed that room temperature increase was better at preventing hyperactivity in ABA than leptin infusion, which led to a decrease in hyperactivity too, but less pronounced (Fraga et al., 2020). Lastly, Hillebrand et al. examined whether a warm plate that was installed in the cage of the rats would influence development of ABA (Hillebrand et al., 2005a). They reported that during ABA, rats preferred the warm plate and hypothermia was prevented, accompanied by reduced hyperactivity and body weight loss when compared to ABA rats without a plate (Hillebrand et al., 2005a).

### Medication Trials in ABA

#### Chlorpromazine

Adams et al. reported that chlorpromazine prevented running induced feeding suppression in rats (Adams et al., 2009). Rats that were treated with the first generation antipsychotic medication, chlorpromazine, had a significantly higher food intake than controls (Adams et al., 2009).

#### Fluoxetine, 8-OH-DPAT and Fenfluramine

Altemus et al. examined drugs with serotonergic effects in ABA (Altemus et al., 1996). They found that ABA rats with daily fluoxetine treatment (SSRI increasing serotonin) ate most and ran least, rats with imipramine (TCA increasing serotonin) ran and ate similar to saline rats and rats with para-chlorophenylalanine (PCPA, tryptophan hydroxylase inhibitor that depletes serotonin) ate least and ran most (Altemus et al., 1996). In another study by Klenotich et al., it was shown that fluoxetine in ABA mice increased food intake and reduced food anticipatory activity, but did not alter survival (Klenotich et al., 2012). Conversely, Atchley et al. found that treatment with 8-OH-DPAT (which reduces serotonergic activity) attenuates weight loss in ABA female rats by reducing hyperactivity (but food intake was similar) (Atchley and Eckel, 2006).

Two studies examined the effects of fenfluramine in ABA: Fenfluramine is an appetite suppressant drug in humans and acts agonistic on 5-HT2C receptors located on pro-opiomelanocortin (POMC) neurons in the arcuate nucleus of the hypothalamus (Atchley and Eckel, 2005; Hillebrand et al., 2006a). Atchley et al. showed that treatment with fenfluramine in female Long Evans ABA rats accelerates the weight loss (Atchley and Eckel, 2005). Hillebrand et al. reported 1 year later in a study in Wistar rats that unexpectedly, fenfluramine treated ABA rats did not reduce food intake or increase wheel running as compared with vehicle-treated ABA rats (Hillebrand et al., 2006a). However, fenfluramine treated ABA rats showed hypodypsia and increased plasma osmolality and arginine-vasopressin expression levels in the hypothalamus (Hillebrand et al., 2006a).

#### Dopamine 2/3 Receptor Antagonists (Antipsychotics)

Rats were chronically infused with the atypical antipsychotic drug olanzapine and exposed to the ABA model or *ad libitum* feeding (Hillebrand et al., 2005c). Olanzapine treatment reduced development of ABA in rats by reducing running wheel activity, starvation-induced hypothermia and activation of the hypothalamus-pituitary-adrenal axis (Hillebrand et al., 2005c). In another study by Klenotich et al. it was demonstrated that olanzapine significantly increased survival and reduced food anticipatory activity in ABA mice (Klenotich et al., 2012). However, olanzapine did not alter food intake or overall running wheel activity (Klenotich et al., 2012).

Additionally, Klenotich et al. showed that D2/3 receptor antagonists eticlopride and amisulpride reduced weight loss and hypophagia and increased survival during ABA in mice (Klenotich et al., 2015). Furthermore, they reported that amisulpride produced larger reductions in weight loss and hypophagia than olanzapine (Klenotich et al., 2015). Treatment with either D3 receptor antagonist SB277011A or the D2 receptor antagonist L-741,626 also increased survival in ABA mice (Klenotich et al., 2015). Verhagen et al. examined the effects of different dosages of treatment with the non-selective dopaminergic antagonist *cis*-flupenthixol in ABA rats (Verhagen et al., 2009a). *cis*-flupenthixol treated ABA rats reduced body weight loss and running wheel activity and increased food intake compared to control ABA rats. Food-anticipatory activity still persists in *cis*-flupenthixol treated ABA rats (Verhagen et al., 2009a).

#### Tetrahydrocannabinol (THC)

Lewis et al. studied the effects of Δ9-THC treatment on ABA. Δ9-THC decreased survival in ABA mice but increased feeding in the survivors (Lewis and Brett, 2010). Contrary findings were reported by Scherma et al.: they showed that subchronic administration of the natural CB1/CB2 receptor agonist Δ9-THC and as well the synthetic CB1/CB2 receptor agonist CP-55,940 decreased body weight loss and running wheel activity in ABA rats (Scherma et al., 2017). Moreover, leptin signaling was increased, while plasma levels of corticosterone were decreased (Scherma et al., 2017). Lastly, the research group by Verty et al. found, too, that Δ9-THC treatment led to an attenuation of ABA in rats (Verty et al., 2011).

#### Clonidine

Rats were implanted subcutaneously with osmotic minipumps infusing 0, 30, or 300 zg/kg/day of clonidine and exposed to ABA (Rieg and Aravich, 1994). Results showed that clonidine did not affect the rate of weight loss during ABA, but increased food intake at the lower dose and wheel activity at the higher dose (Rieg and Aravich, 1994).

### Recovery From ABA

#### Refeeding After ABA With Continuous Access to Running Wheel

Achamrah et al. examined the effects of voluntary access to a running wheel during refeeding of ABA mice and found that exercising in a running wheel during refeeding had positive effects (Achamrah et al., 2016b). Only the mice with access to running wheels completely restored their fat-free mass from before having undergone ABA and had less colonic hyperpermeability than their sedentary refeeding controls (Achamrah et al., 2016b). The general locomotor behavior and specifically the behavior in dark-light boxes of the mice that ran in running wheels improved compared to the sedentary controls (Achamrah et al., 2016b).

#### Taste Aversion Learning

Liang et al. compared the acquisition and extinction of a conditioned taste aversion in a naïve, ABA, and pair-fed rat group (Liang et al., 2011). The conditioned taste aversion conditioning was done after the ABA and pair-fed rats had regained all their weight from before the food restriction (Liang et al., 2011). For the CTA learning, 0.3 M sucrose consumption was followed by low doses lithium chloride (0.009 M or 0.018 M at 1.33 ml/100 g of body weight, ip) injection (Liang et al., 2011). The results showed that the ABA rats developed an aversion to sucrose significantly sooner than the naïve controls (Liang et al., 2011). Furthermore, their response was more persistent, as they completely avoided sucrose, while the naïve and pair-fed controls still tried it by the end of 10 conditioning trials (Liang et al., 2011). When extinction was assessed by 1-bottle and 2-bottle tests, it took the ABA rats longer to extinguish the aversion than the controls (Liang et al., 2011).

#### Neuroendocrine Changes

Mequinion et al. investigated what effects long-term energy deficits in mice exert after recovery of body weight (Mequinion et al., 2017). They showed that after a long-term energy deficit (10 weeks), mice exhibited persistent hypoleptinemia following the refeeding period (10 weeks) despite restoration of fat mass, ovarian activity, and feeding behavior (Mequinion et al., 2017). The refeeding period induced an overexpression of leptin receptor mRNA in the hypothalamus (Mequinion et al., 2017).

## Discussion

We performed a systematic literature review of studies on animal models for anorexia nervosa. The research on animal models for anorexia nervosa has already started decades ago and many findings contributed to the understanding of mechanisms of hunger and satiety, physical activity and cognition in an underweight state and other mechanisms relevant for anorexia nervosa in humans.

Different animal models of anorexia nervosa have been developed with advantages and disadvantages. In our review, we found and described 18 different animal models. One model of particular interest from among the genetic animal models is the anx/anx model. Interesting findings about the effects of hunger/satiety regulatory peptides like neuropeptide Y and agouti-related peptide have been made in this model. An advantage of the model is that despite the animals having *ad libitum* access to food, they still lose body weight, which mimics one feature of anorexia nervosa. A disadvantage of the model is that animals have many other neurological changes apart from hypophagia and overall have a very short survival time, therefore many researchers have concluded that their genetic expression profile along with the phenotype resembles more cachexia syndromes observed in cancer or chronic diseases rather than anorexia nervosa (Mercader et al., 2008).

The most commonly used animal model to study anorexia nervosa is the activity-based anorexia (ABA) model. It does mimic some aspects of anorexia nervosa, namely, body weight loss, increased physical activity, cessation of the estrous cycle in females and alterations in the hypothalamus-hypophysis-adrenal axis. The most frequent points of critique were the lack of psychosocial factors that play a decisive role in patients with anorexia nervosa and the acuity of the model that does not reflect the human condition that often starts in adolescence but might go on for many years. The chronic ABA models developed by Frintrop et al. for rats (Frintrop et al., 2018b) and by Mequinion et al. for mice (Mequinion et al., 2015b) do provide a more chronic time course than the acute ABA model and therefore reflect better the human condition. Two factors that are still not reflected in this model are that genetic predisposition makes some humans more susceptible to anorexia nervosa and that psychosocial factors play a role in the first manifestation and during the course of the disease.

Specific neurocognitive and behavioral changes were observed in the ABA model, including changes in anxiety-like behavior in which decreased estrogen might lead to increased anxiety (Kinzig and Hargrave, 2010; Campos et al., 2019). Apart from that an individual rat's level of anxiety behavior might correlate with its physical activity in the running wheel (Campos et al., 2019). Rats' baseline physical activity in a cage with and without a running wheel during the *ad libitum* fed period, is the best predictor for a rat's individual chance of susceptibility to ABA (Perez-Leighton et al., 2014). One study examined effects of stool transplantation of anorexia nervosa patients in germ free mice in their female offspring and the results are very interesting and warranting further research (Hata et al., 2019). Intestinal barrier alterations are observed in ABA including increased colonic permeability (Jesus et al., 2014). Neuroendocrine changes that were described in the ABA model include an increase in α4 GABA receptors in the CA1 hippocampus and amygdala (Aoki et al., 2012; Wable et al., 2014), alterations and decreased cell proliferation in the hippocampus (Chowdhury et al., 2013a) and a reduction in the density of astrocytes and a reduction in the volume of the cerebral cortex and corpus callosum (Frintrop et al., 2018a), which might be completely reversible by refeeding (Frintrop et al., 2019). Other findings were altered endocannabinoid (Casteels et al., 2014), histaminergic (Endou et al., 2001), BDNF (Gelegen et al., 2008; Ho et al., 2016), and dopaminergic (Gelegen et al., 2008; Gilman et al., 2019) transmission. Several studies investigated the effects of medication on ABA where specifically chlorpromazine (Adams et al., 2009), fluoxetine (Altemus et al., 1996), olanzapine (Klenotich et al., 2012), amisulpride (Klenotich et al., 2015), and cis-flupenthixol (Verhagen et al., 2009a) were shown to reduce ABA symptoms and point therefore in future directions of medication trials in anorexia nervosa.

There are some limiting factors to consider for this systematic review. First, by searching only from among the three most commonly used scientific databases we likely exclude some studies (e.g., so-called gray literature). These databases sometimes do not have the best fitting mesh/keywords for articles, so that many articles were found in our search that we were not looking for, that were for example human studies or animal models on other diseases than anorexia nervosa and these had to be excluded. Additionally, papers were not included that did not show up in the search. Second, we had strong exclusion criteria that reduced the total number of included studies to 108. We may have therefore excluded additional information that might have been found in the excluded reviews, including articles written in another language.

Despite the shortcomings of animal models in general and models for anorexia nervosa in particular, we do encourage the further investigation of animal models for anorexia nervosa. As we have explained above, we especially recommend future research in the chronic ABA model that is a further development of the ABA model. Although many aspects of anorexia nervosa remain poorly understood, many finding of studies discussed in this review present interesting directions toward further research. We encourage especially further research on the gut-brain-axis including intestinal microbiome changes and further research in neuronal brain circuit alterations including neurotransmitter changes.

## Data Availability Statement

The original contributions presented in the study are included in the article/supplementary material, further inquiries can be directed to the corresponding author/s.

## Author Contributions

SS performed the literature search and wrote the first draft of the paper. AS planned and supervised the project as well as thoroughly revised the paper. All authors contributed to the article and approved the submitted version.

## Conflict of Interest

The authors declare that the research was conducted in the absence of any commercial or financial relationships that could be construed as a potential conflict of interest.

## Abbreviations

5HT4: serotonin receptor 4
ABA: Activity-based anorexia
BDNF: brain derived neurotrophic factor
CART: Cocaine- and amphetamine-regulated transcript
CTA: conditioned taste aversion
GABA: Gamma aminobutyric acid
GH/IGF-1 axis: growth hormone/insulin-like growth factor 1 axis
HPA axis: hypothalamus pituitary adrenal axis
icv: intracerebroventricular
ip: intraperitoneal
NAc: Nucleus accumbens
NMDA: N-Methyl-D-Aspartat
POMC: Pro-opiomelanocortin
THC: tetrahydrocannabinol
zg: zeptogram.
